# Relationship of the metabolic syndrome to carotid ultrasound traits

**DOI:** 10.1186/1476-7120-4-28

**Published:** 2006-07-07

**Authors:** Rebecca L Pollex, Khalid Z Al-Shali, Andrew A House, J David Spence, Aaron Fenster, Mary Mamakeesick, Bernard Zinman, Stewart B Harris, Anthony JG Hanley, Robert A Hegele

**Affiliations:** 1 Robarts Research Institute, London, Ontario, Canada; 2 Department of Medicine, University of Western Ontario, London, Ontario, Canada; 3 Sandy Lake Health and Diabetes Project, Sandy Lake, Ontario, Canada; 4 Department of Medicine, University of Toronto, and Samuel Lunenfeld Research Institute, Mount Sinai Hospital, Toronto, Ontario, Canada; 5 Thames Valley Family Practice Research Unit, University of Western Ontario, London, Ontario, Canada; 6 Department of Nutritional Sciences, University of Toronto, Toronto, Ontario, Canada

## Abstract

**Background:**

The metabolic syndrome is associated with increased vascular disease risk. We evaluated two carotid ultrasound measurements, namely intima media thickness and total plaque volume, in a Canadian Oji-Cree population with a high metabolic syndrome prevalence rate.

**Methods:**

As part of the Sandy Lake Complications Prevalence and Risk Factor Study, 166 Oji-Cree subjects (baseline metabolic syndrome prevalence, 44.0%, according to the National Cholesterol Education Program Adult Treatment Panel III guidelines) were examined using a high-resolution duplex ultrasound scanner.

**Results:**

Image analysis showed that mean intima media thickness was elevated in subjects with the metabolic syndrome (818 ± 18 *vs *746 ± 20 μm), as was total plaque volume (125 ± 26 *vs *77.3 ± 17.0 mm^3^). However, after adjustment for age and sex, the differences were significant only for intima media thickness (*P *= 0.039). Furthermore, a significant trend towards increased intima media thickness was observed with increasing numbers of metabolic syndrome components: mean intima media thickness was highest among individuals with all five metabolic syndrome components compared to those with none (866 ± 55 *vs *619 ± 23 μm, *P *= 0.0014). A similar, but non-significant trend was observed for total plaque volume.

**Conclusion:**

This is the first study of the relationship between the metabolic syndrome and two distinct carotid ultrasound traits measured in the same individuals. The results suggest that standard intima media thickness measurement shows a more consistent and stronger association with the metabolic syndrome than does total plaque volume.

## Background

The metabolic syndrome (MetS) is linked to increased levels of vascular damage [1], cardiovascular morbidity and cardiovascular mortality [2-4]. A survey of the most recent evidence estimated that cardiovascular disease risk is increased ~2-fold in both men and women with MetS [2]. Non-invasive carotid ultrasound techniques can potentially be used to test the validity of this relationship at a much earlier pre-clinical stage. Furthermore, these surrogate measurements of cardiovascular disease may assess a patient's risk and be used in the evaluation of novel treatments [5].

The commonly used measurement to assess carotid "atherosclerosis" is intima media thickness (IMT). Recently, however, newer measurements such as assessment of plaque area or total plaque volume (TPV) have been suggested to represent a potentially more powerful approach, since these measurements in higher dimensions evaluate plaque burden in the carotid system and hold great sensitivity and discrimination [6-8]. IMT and TPV, however, are not interchangeable. Their correlation is only moderate (*r *< 0.7) and these different ultrasound-derived measures of carotid artery morphology likely represent distinct attributes of atherosclerosis [9,10]. As more research is needed to provide a complete understanding of how these ultrasound traits relate to cardiovascular disease stages and endpoints we have evaluated two carotid ultrasound measurements, namely IMT and TPV, in a Canadian Oji-Cree population with one of the highest MetS prevalence rates in the world [11].

## Methods

### Study subjects

All subjects in the current study had formerly been participants in the original 1993–1995 Sandy Lake Health and Diabetes Project [12]. The Oji-Cree community of Sandy Lake, Ontario, is located ~2000 km northwest of Toronto, in the subarctic boreal forest of central Canada. 728 members of this community (72% of the total population), ≥10 years of age, participated in the original survey. Detailed information on demographics, dietary habits, and physical fitness of the study participants has been previously reported [13-15]. In a separate study initiated in 2001, the Sandy Lake Complications Prevalence and Risk Factor Study [16], 278 subjects (51.1% with diabetes) who were free of coronary heart disease had ultrasound assessment of the carotid arteries. The studies were approved by the Sandy Lake First Nation Band Council and the University of Toronto Ethics Review Committee. For this study, 166 subjects were selected on the basis of having both a complete set of data for MetS diagnosis from the original survey and carotid ultrasound measurements from the follow-up study. Signed informed consent was obtained from all participants.

### Clinical characteristics and biochemical analysis

Body weight, height, waist circumference, and blood pressure were measured by standardized procedures [12]. Hypertensive individuals were defined as subjects having increased blood pressure (≥130 mmHg systolic and/or ≥85 mmHg diastolic) or on antihypertensive drug treatment. Measurements of fasting blood analytes, including triglycerides, glucose, and lipoproteins were performed as described [12].

### Metabolic syndrome (MetS) case definition

According to the National Cholesterol Education Program Adult Treatment Panel III (NCEP ATP III) criteria [17], MetS was identified if a subject had ≥3 of: 1) increased waist circumference (>102 cm [>40 inches] for men, >88 cm [>35 inches] for women); 2) elevated plasma triglycerides (≥1.69 mmol/L [≥150 mg/dL]); 3) low plasma HDL cholesterol (<1.04 mmol/L [<40 mg/dL] for men, <1.29 mmol/L [<50 mg/dL] for women); 4) increased blood pressure (≥130 mmHg systolic and/or ≥85 mmHg diastolic) or on antihypertensive drug treatment; and 5) impaired fasting glucose (≥6.1 mmol/L [≥110 mg/dL]).

### Ultrasound examination

Subjects were examined using a high-resolution duplex ultrasound scanner HDI 5000 equipped with Sono-CT compound imaging and a L12-5 transducer (Advanced Technology Laboratories, Bothell, Washington) that had been flown to the community and housed within the Diabetes Research Center. Common carotid ultrasound images for all participants were gathered over a 4-week period, and from this data, intima-media thickness (IMT), total plaque area (TPA), and total plaque volume (TPV) measurements were determined [9,10]. TPA was strongly correlated with TPV in these subjects (*r *= 0.921, *P *< 0.0001) and thus for simplicity, only TPV measurements were used. The correlation between IMT and TPV was 0.467 (*P *< 0.0001).

### IMT measurement

IMT was determined as previously described [9,10]. Briefly, a single observer, blinded to subjects' vascular risk, measured combined thickness of intima and media of the far wall of both common carotid arteries. Images were recorded from an anterolateral longitudinal view. The still images were analyzed using computerized edge-detection software (Prowin™) [18]. Using a step-wise algorithm, conditional sets of "edges" (consisting of lumen-intima and media-adventitia echoes) were located within the image and then tested for "edge strength", with the subsequent deletion of weak edge points. Once all acceptable edge points were identified, boundary gaps were filled by linear interpolation. The distance between lumen-intima and media-adventitia boundaries was then measured to calculate IMT. Mean IMT was computed from 120 measurements over a 10 mm span ending 5 mm proximal to the transition between the common carotid and bulb regions. Intra- and inter-operator coefficients of variation were 3.0 and 3.1%, respectively, and intra- and inter-operator intraclass correlations were both 0.97.

### TPV measurement

TPV was determined as previously described [9,10]. Briefly, 3D ultrasound images were acquired using a freehand scanning system and analyzed with L3Di visualization software (Life Imaging Systems Inc., London, Ontario). Plaque volumes were measured using manual planimetry: each 3D image was 'sliced' transversely at an inter-slice distance of 1 mm, moving from one plaque edge to the other. Plaque boundaries were traced using a mouse driven cross-haired cursor. Slice areas were summed and multiplied by inter-slice distance to calculate plaque volume. For this analysis, TPV was defined as the sum of all plaque volumes between the clavicle and angle of the jaw for both carotids, including areas from both the common and internal carotid systems. Intra- and inter-operator coefficients of variation were 6.5 and 6.9%, respectively, and intra- and inter-observer reliability were 0.87 and 0.91, respectively [19].

### Statistical analysis

SAS version 8.2 (SAS Institute, Cary, North Carolina) was used for all analyses. The FREQ procedure was used to determine the frequencies of categorical variables. Differences in proportions were tested by χ^2 ^analysis. The mean ± standard error (SE) of continuous variables describing the demographic and laboratory characteristics for those with and without MetS were analyzed by ANOVA, using the general linear model, adjusting for age and sex. Plasma triglycerides were not normally distributed and were log transformed. Significance was calculated from type III sums of squares, which are most appropriate for unbalanced study designs and report significance after all covariates are taken into account. Differences in IMT and TPV were also analyzed using the general linear model, adjusting for age and sex. IMT and TPV were both significantly non-normal in this dataset and thus were transformed using the inverse of IMT and the square root of TPV. Pair-wise differences in IMT or TPV according to the number of MetS components were compared using the t-statistic for least squares means from ANOVA. Statistical significance was taken at nominal *P *< 0.05 for all comparisons. Transformed values were used for statistical comparisons, but untransformed values are shown in the tables and figures.

## Results

### Demographics, clinical and biochemical features based on MetS

Table 1 shows the demographic and metabolic characteristics of Oji-Cree subjects with and without MetS. MetS prevalence was 44.0% (73/166) according to the NCEP ATP III criteria. Those with MetS were significantly older than those without MetS (*P *= 0.0019); no differences were found with respect to sex and smoking habits. As expected, clinical and biochemical measurements that contributed to the MetS definition, such as waist circumference, HDL cholesterol, triglycerides, blood pressure, and fasting glucose were significantly different between subjects with and without MetS. In addition, other traits not considered to be a part of the official MetS definition, such as BMI (body mass index), total cholesterol and TC:HDL ratio were also elevated in the MetS subjects (*P *< 0.0001).

**Table 1:** Demographic and clinical features of Oji-Cree study group

**Characteristic**	**MetS present N = 73**	**MetS absent N = 93**	***P*-value adjusted for age and sex**
age (years)	42.2 ± 1.6	34.8 ± 1.6	0.0019
male (%)	35.6	43.0	NS (0.33)
current smokers (%)	13.9*	16.3†	NS (0.86)
BMI (kg/m^2^)	31.3 ± 0.5	27.4 ± 0.5	<0.0001
waist (cm)	104 ± 1	92.9 ± 1.2	<0.0001
hypertensive (%)	54.8	18.3	<0.0001
antihypertensive treatment (%)	19.2	5.4	0.017
total cholesterol (mmol/L)	5.21 ± 0.10	4.38 ± 0.08	<0.0001
HDL cholesterol (mmol/L)	1.09 ± 0.03	1.27 ± 0.02	<0.0001
triglycerides (mmol/L)	2.36 ± 0.11	1.24 ± 0.05	<0.0001
TC:HDL ratio	4.99 ± 0.15	3.54 ± 0.09	<0.0001
fasting glucose (mmol/L)	9.48 ± 0.54	6.34 ± 0.30	<0.0001
diabetes (%)	67.1	17.2	<0.0001
IGT (%)	8.2	14.0	NS (0.72)

*N = 72, †N = 92

Data are means ± SE, unless otherwise stated.

Abbreviations: IGT, impaired glucose tolerance; NS, not significant

### Relationship with carotid atherosclerosis

**Figure 1:**
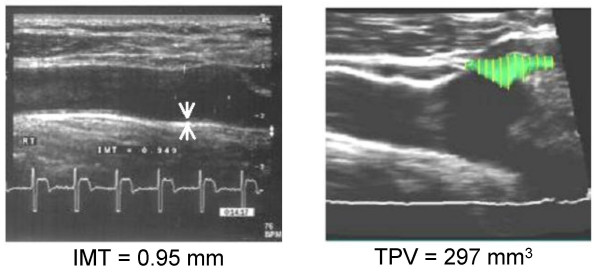
Ultrasound images used for the determination of carotid anatomy in a subject with MetS. The panel on the left shows an image of the right carotid artery used to determine intima media thickness (IMT), with the arrows at the far carotid wall showing where IMT was determined. The panel on the right shows an image used to determine total plaque volume (TPV), with the encircled coloured region defining one of the plaques identified.

Carotid ultrasound measurements were gathered 7 years following the baseline MetS diagnosis (Figure 1). As shown in Table 2, values for both mean IMT and TPV were elevated in those with MetS. However, after adjustment for age and sex, the observed differences were significant only for the evaluation of IMT (818 ± 18 [MetS present] *vs *746 ± 20 μm [MetS absent], *P *= 0.039).

**Table 2:** Carotid ultrasound measurements according to presence or absence of MetS

**Trait**	**MetS present N = 73**	**MetS absent N = 93**	***P *unadjusted**	***P *adjusted for age and sex**
Mean IMT (μm)	818 ± 18	746 ± 20*	0.0008	0.039
TPV (mm^3^)	125 ± 26	77.3 ± 17.0	0.021	NS (0.64)

*N = 91

Data are means ± SE.

**Figure 2:**
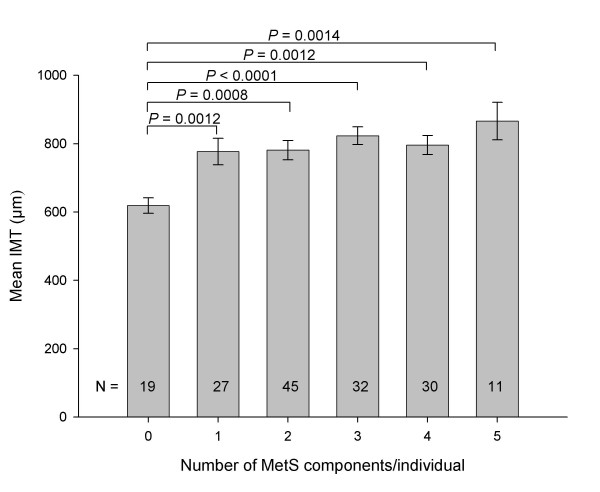
Mean carotid IMT values, plotted according to an individuals' number of MetS components (mean ± SE).

**Figure 3:**
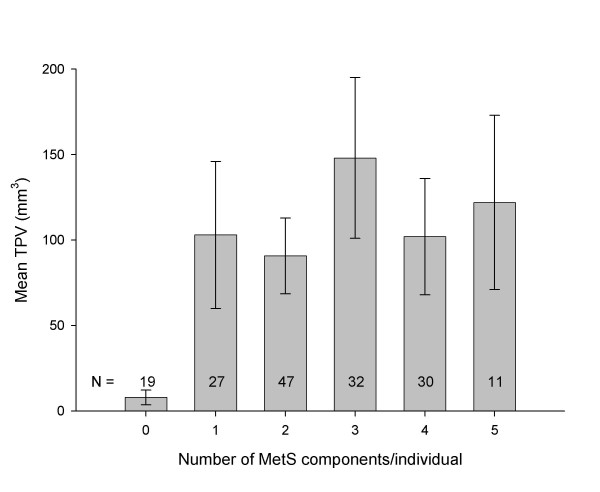
Total carotid plaque volume, plotted according to an individuals' number of MetS components (mean ± SE). All *P*-values for pair-wise differences in TPV were not significant (*P *> 0.05).

Evaluations based on the number of MetS components present showed a significant trend towards increased IMT with increasing numbers of MetS components (Figure 2). Those with all five MetS components had the greatest IMT while those with none of the components had the smallest IMT (866 ± 55 *vs *619 ± 23 μm, *P *= 0.0014). Each pair-wise comparison of increasing MetS component number was significant. In contrast, for TPV, no significant differences were observed between individuals with any number of MetS components and those with none (Figure 3).

## Discussion

We found: 1) elevated carotid IMT for subjects with MetS in comparison to those without MetS (*P *= 0.039); 2) no significant difference in carotid TPV for subjects with MetS, adjusting for age and sex; and, 3) a trend towards increasing IMT with increasing numbers of MetS components. The results suggest that IMT has a stronger and more consistent relationship with MetS than does TPV.

Our results are consistent with previous studies of IMT which have also found a significant association between increased carotid artery thickening and MetS diagnosis. In the largest study to date, the Atherosclerosis Risk in Communities (ARIC) study (N = 12,178), MetS was significantly associated with both an increased risk of coronary heart disease and also with increased IMT. The observed difference between those with and without MetS was relatively small (747 *vs *704 μm), but highly significant (*P *< 0.0001), even after adjustment for age, sex, and race/study [20]. Similarly in an Austrian study (N = 1,588), IMT was found to be significantly higher in subjects with MetS (*P *< 0.0005) [21]. The observed difference in IMT between those with and without MetS was more pronounced in females than in males, indicating a potentially stronger relationship between MetS and atherosclerosis in females [21].

One distinctive feature of our study was the relatively younger age of our Oji-Cree sample, which may be affected by a lesser degree of measurable disease progression. Interestingly we observed a difference in IMT of ~70 μm between those with and without MetS, greater than the difference observed in the ARIC study, yet similar to the values observed for the middle-aged females in the Austrian study. Observing this degree of IMT for subjects with MetS among the relatively young Oji-Cree collective is consistent with the high prevalence of both type 2 diabetes and coronary heart disease that have been observed in this population [14,22].

While a significant difference was found for IMT, no significant difference was found for TPV between subjects with MetS and subjects without MetS, although TPV tended to be greater among subjects with MetS. The absence of statistical significance following adjustment for age and sex is undoubtedly related to the small number of subjects and the proportion of individuals without measurable plaque (TPV ≤ 0.01 mm^3 ^for 31% of subjects), but it may also be because of the relative insensitivity of carotid TPV as a surrogate marker for atherosclerosis (i.e. greater intra- and inter- operator variation) or because patients with MetS have increased IMT rather than increased plaque. Previous studies have reported that the correlation between IMT and TPV is only modest (*r *< 0.7), indicating that these measures likely represent distinct attributes of atherosclerosis [7,9,10]. This is understandable, considering that the measurement of IMT is based on a single defined location near to the transition between the common carotid and bulb regions, whereas the measurement of TPV involves the whole carotid system, and thus disparities can arise depending on whether or not plaque happens to be present in this designated area. When measured in the same subjects, these ultrasound traits have been shown to have distinct relationships with individual risk factors [9], diabetes [7], and genetic determinants [23,24]. TPV may better reflect the later stages of plaque formation and the total disease burden, especially among subjects with diabetes [7,9], whereas IMT, correlating more closely with hypertension and age [9], may reflect wall hyperplasia or hypertrophy related to hypertension. This considered, it may be that IMT would capture vascular disease burden more effectively in MetS-affected subjects, considering that both systolic and diastolic blood pressure are key components of the MetS definition.

A limitation of our study and others is the lack of prospective data to determine the relationship between MetS and the progression of carotid ultrasound traits. A prospective study of 316 Swedish middle-aged men found that IMT was greater at baseline and also after three years in subjects with MetS, while no significant change was observed for subjects with no MetS risk factors [25]. Further prospective studies such as these, analyzing the use of ultrasound measures for detecting disease progression, would be potentially valuable for future MetS research.

In addition, it would have been beneficial if our study evaluated additional, more general populations, to determine if this pattern of positive association for IMT and no association for TPV is typical or simply a unique observation for the Oji-Cree. The Oji-Cree study group was also not just a typical population, in that the prevalence of diabetes was >50% for the MetS subjects. Considering the distinctiveness of the carotid ultrasound trait measures, it is indeed highly likely that there will also be distinct relationships observed depending on the population studied.

## Conclusion

Our study of the relationship between MetS and carotid atherosclerosis has, to the best of our knowledge, evaluated IMT and TPV in parallel, for the first time. For this study group, the observations show that standard IMT measurement captures an increased disease burden among individuals with MetS, while the TPV measurement appears somewhat less strongly related and less informative. This supports previous reports of increased carotid ultrasound analytes for persons with MetS, but additionally, this study indicates that TPV may not be as valuable a surrogate marker of vascular disease burden in MetS, though this is likely dependent on the characteristics of the study population.

Ultrasound measurements are, however, only surrogate markers for risk of arterial occlusion. What would be of great interest is to know which test correlates more closely with outcome. This is an important step for future studies. Though increased TPV was not a significant hallmark for those with MetS in this study, it still, nonetheless, is a distinct ultrasound trait which may be shown to be an important preclinical marker for arterial occlusion risk.

## Competing interests

The author(s) declare that they have no competing interests.

## Authors' contributions

RLP participated in the design of the study, analysis of the data, and writing of the manuscript. KZA and AAH calculated the total plaque volume and intima-media thickness measurements, respectively, and assisted with manuscript revisions. MM coordinated the data collection. JDS, AF, BZ, SBH, and AJGH provided patients and data for the study, and assisted with manuscript revisions. RAH participated in the design of the study and writing of the manuscript. All authors read and approved the final manuscript.
